# Annotations

**Published:** 1905-07-29

**Authors:** 


### ANNOTATIONS.

### Proposed Union of London Medical Societies.

The report of the committee appointed at a representative meeting of the profession held in April of the present year to discuss the possibility of a union or fusion of the existing medical societies is a serious and business-like document. The question has evidently been considered in a reasonable and practical fashion, and there is now good ground for anticipating that the desired end will be attained. It is proposed that a new society shall be formed and shall be called either the Royal Society of Medicine or the Royal Academy of Medicine. This, it is suggested, shall include sixteen sections, which will correspond fairly accurately with the existing societies representing the various phases of professional activity and interest. One result would be the fusion of duplicate societies such as exist to-day in the departments of dermatology and laryngology. It is also proposed to join in a common section, to be named "Therapeutical," the departments now cultivated by the Therapeutic, Electro-therapeutic, and Balneological and Climatological Societies. The fees suggested are a payment of three guineas annually by each Fellow of the new Society or Academy, but membership of any one of the sections will be open for an annual subscription of one guinea, and of a second section for an additional half-guinea. It is estimated that these charges will permit the publication during nine months of the year of the *Proceedings* of the Society and of an annual volume of selected papers as *Transactions*, as well as provide for working expenses with a balance for improvements and a sinking fund. These estimates are framed upon an investigation conducted by a firm of accountants into the income and expenditure of the existing twenty-two London societies, and do not include any saving which may result in working should the union be accomplished. There thus appears to be a sound business basis for the new proposal, the professional value of which is generally recognised. The spirit in which the negotiations have been conducted appears to have been admirable, and affords fair promise of a successful issue.

### Diplomacy and Medicine.

An interesting incident of the proceedings at the fourth centenary of the Royal College of Surgeons, which was held at Edinburgh last week, was a speech delivered on Friday by the French Ambassador at the luncheon given under the presidency of Dr. John Playfair. M. Cambon, who was invited to respond to the toast of "The Guests," modestly protested that in such an assembly of distinguished men of science he would not have had the right to express their sentiments of gratitude if he had not known that a comparison had been sometimes made between the rôle of the physician and that of the diplomatist. While he agreed that the medical profession had to alleviate suffering, and to fight death, he claimed for diplomatists that they had to avoid between nations those frictions which degenerated into inflammation, and sometimes more seriously affected the organisms of peoples; and he repudiated the theory of "some evil-minded persons" that if there were no diplomatists there would be no wars. M. Cambon also congratulated the medical profession upon the advantage they enjoy over diplomatists "because the results they obtain are clear to all eyes, whereas those achieved by diplomatists are hidden from the appreciation of contemporary community." But has His Excellency never heard the theory of "some evil-minded persons" that if there were no medical men there would be no diseases? The members of the medical profession do not complain of want of appreciation, or even of misrepresentation. But we think that they are at least as liable to the one and as much exposed to the other as diplomatists. If, too, the actual part played by diplomatists in averting, or striving to avert, conflicts between nations is not revealed to the world at the time, there are such palliations, at least in this country, as large salaries, promotions, and titles, which have always been given with no niggard hand to these useful and favoured servants of the State. Assuming that the advantages attending the pursuit of diplomacy and medicine are weighed only in regard to passing advantages, we fancy that a less cursory comparison than that of M. Cambon would not tempt well-equipped young men in search of a profession to choose that of medicine.

### The Birth-rate and the Working Classes.

In a recent issue we drew attention to the low level of the birth-rate in a fashionable West-end district as indicative of the growing disinclination on the part of the more prosperous classes to accept the full responsibilities of married life. A similar state of matters is attracting attention on the other side of the Atlantic, as is evidenced in a volume just published by Dr. Hugo Münsterberg, professor of psychology at Harvard. In this, allusion is made to the objection shown by many cultured women to the demands of family life, and their even more pronounced objection to bear children. The conclusion inevitably suggested is that future generations in the United States will contain a decreasing proportion of the cultured classes and a growing percentage of the descendants of those who reach the country as immigrants, and who, for the most part, represent a lower social and educational level. It may be questioned, however, whether the responsibilities and opportunities of parentage are being shirked only by the idle and luxurious. In a paper circulating largely amongst the working classes, and a copy of which is now lying before us, we find some half-dozen advertisements which, either in a concealed or open fashion, announce an ability on the part of the advertiser to supply either information or apparatus by which conception may be prevented. All this argues a considerable decline in the estimate of what is decent and becoming in the columns of a public newspaper. But, more seriously still, it indicates how widespread is the disinclination to accept the burden of family responsibilities. The effect of this on the interests and future of the nation can hardly be exaggerated, and the responsibility of those who further such ends is not an enviable one. So far as a practical solution is possible it must be found in a robust assertion of public opinion. It lies with the press, and with teachers who command public confidence, not to shirk the difficulties of the situation.

